# Effect of static stretching duration on modulation of H‐reflex and tendon‐reflex excitability of the soleus muscle in young men

**DOI:** 10.14814/phy2.70538

**Published:** 2025-08-26

**Authors:** Akira Saito, Takamasa Mizuno

**Affiliations:** ^1^ Center for Health and Sports Science Kyushu Sangyo University Fukuoka Japan; ^2^ Research Center of Health, Physical Fitness and Sports Nagoya University Nagoya Japan

**Keywords:** ankle joint, flexibility, muscle spindle sensitivity, spinal excitability

## Abstract

Stretching‐induced impairments of muscle performance are attributed to neural adaptations and mechanical changes. Inhibition of muscle spindle sensitivity appears to have long‐lasting effects after stretching. However, whether a dose–response relationship exists between stretching duration and muscle spindle sensitivity remains unclear. The present study aimed to reveal the effect of static stretching duration on modulation of muscle spindle sensitivity in the soleus muscle. The present study used data obtained from 19 young men. Static stretching intervention involved five 1‐min stretches with 1‐min intervals between stretches under maximal dorsiflexion. The Hoffmann‐reflex (H‐reflex) and tendon‐reflex (T‐reflex) were recorded from the soleus before and during stretching, at intervals between stretching. Time‐course changes in H‐reflex and T‐reflex amplitudes from baseline (i.e., before stretching) were calculated. The H‐reflex amplitude depressed 51.5%–55.2% during static stretching, and these H‐reflex depressions recovered in the interval following each stretch. T‐reflex amplitudes depressed 71.2%–73.5% during static stretching, and these T‐reflex depressions remained following each interval. Inhibitions of the T‐reflex amplitude after the second to fifth stretches were not significantly stronger than that after the first static stretch. These results suggest that 1‐min static stretching under maximal dorsiflexion achieves sufficient modulation of muscle spindle sensitivity of the soleus.

## 1 INTRODUCTION

Maximal joint range of motion (ROM) and passive torque to stretch (indices of stiffness and stretch tolerance) are functional parameters that may affect the risk of muscle strain injury (Witvrouw et al., 2003). Although static stretching is commonly used by athletes and in clinical settings to acutely improve ROM and decrease stiffness of the muscle‐tendon unit, the acute effects of static stretching have been suggested to impair muscle strength (Behm et al., 2016; Behm & Chaouachi, 2011; Kay & Blazevich, 2012). Impairment of muscle strength after static stretching is caused by both neural and mechanical changes, and these effects have been suggested to remain several minutes after static stretching. However, the acute effects of static stretching in terms of the modulation of neural circuits are not fully understood. In most cases, static stretching involves a warm‐up routine and is performed prior to physical exercise and athletic events (Woods et al., 2007). Clarification of stretching‐induced specific neural pathways would thus help reveal the mechanisms by which static stretching modifies ROM and muscle performance.

Previous studies have shown that Hoffmann‐reflex (H‐reflex) responses in the soleus (SOL) muscle are inhibited during static stretching, and this depression of H‐reflex responses immediately returns to the baseline state seen prior to stretching (Avela et al., 1999; Funase et al., 2003; Guissard et al., 1988; Obata et al., 2022). Such results suggest that changes in muscle length during static stretching suppress spinal reflex excitability. Inhibitory mechanisms that mediate spinal reflex excitability during static stretching are involved in the modulation of afferent inputs from intramuscular receptors (e.g., muscle spindles) (Guissard & Duchateau, 2006) and presynaptic inhibition (Funase et al., 2003; Guissard et al., 2001). Tendon‐reflex (T‐reflex) responses elicited by tendon tap are influenced by the modulation of spinal reflex excitability and by changes in the sensitivity of the muscle spindles (Guissard & Duchateau, 2006; McNeil et al., 2013). Due to differences in the neural pathways used by the H‐reflex and T‐reflex, depression of the T‐reflex is greater than that of the H‐reflex during static stretching (Budini et al., 2017; Guissard et al., 1988). Interestingly, inhibition of the T‐reflex seems susceptible to a long‐lasting effect and recovery requires 5–10 min following static stretching (Budini et al., 2017, 2019). This long‐lasting inhibition after static stretching is suggested to be caused by reduced sensitivity of the muscle spindle, due to increased compliance of the intrafusal muscle fibers (Avela et al., 1999; Proske et al., 1993). The decreased stiffness of the muscle‐tendon unit induced by static stretching could result in a reduced responsiveness of muscle spindles, which then induces a reduction in Ia afferent inputs onto motoneurons. However, the time course of the modulation of muscle spindle sensitivity by static stretching remains unclear.

As an underlying technical factor in static stretching, the duration of the stretch influences the impairment of muscle strength (Behm & Chaouachi, 2011; Kay & Blazevich, 2012). A curvilinear dose–response relationship has been demonstrated between stretching duration and reduction of muscle performance, and the dose–response effect is independent of the type of task (i.e., strength‐, power‐, and speed‐dependent tasks) and muscle group (Kay & Blazevich, 2012). A previous study showed that H‐reflex and T‐reflex amplitudes in the SOL were depressed with stretching for 30 s, and these depressions did not change with 10 min of continuous stretching (Guissard & Duchateau, 2006). However, that study examined time‐course changes in the H‐reflex and T‐reflex with a 10‐min stretch at maximal dorsiflexion. These modulations might thus have involved changes in muscle length and reflex muscle activity of the lower leg during stretching. In addition, intermittent stretching has been shown to exert greater effects on the magnitude and duration of muscle strength loss than continuous stretching (Trajano et al., 2014). The present study therefore evaluated the modulation of H‐reflex and T‐reflex excitability during static stretching performed under an intermittent protocol at a constant joint position, to test whether a dose–response effect on muscle spindle sensitivity is observed with static stretching.

The purpose of the present study was to reveal the effect of static stretching durations on the modulation of muscle spindle sensitivity in the SOL. We hypothesized that inhibition of T‐reflex excitability would be enhanced with increasing duration of stretch. To test this hypothesis, we examined the effect of the number of repetitions of a 1‐min static stretching on H‐reflex and T‐reflex excitability in the SOL. Moreover, to identify the temporal effects of long‐lasting inhibition of muscle spindle sensitivity with static stretching intervention, further measurements were performed until 20 min after the stretching intervention.

## 2 MATERIALS AND METHODS

### 2.1 Subjects

Twenty‐four men were recruited to the present study. T‐reflex responses of the SOL were unable to be evoked from five of the 24 subjects using a handmade reflex hammer. Consequently, the present study used data from the remaining 19 healthy men (values are given as mean ± standard deviation [SD]; age, 19.1 ± 1.0 years; height, 170.8 ± 6.0 cm; body mass, 63.5 ± 7.2 kg). No subjects had any recent history of lower limb musculoskeletal injuries or neuromuscular disorders. The procedure, purpose, risks, and benefits associated with the present study were explained to each subject, and written, informed consent was obtained from each prior to enrollment.

### 2.2 Study design

Subjects visited the laboratory on three occasions, with an interval between each visit of at least 48 h. The first visit involved a familiarization trial, and the subsequent two visits assessed the effect of static stretching on the spinal reflex circuits of the SOL. One experiment was performed to test the H‐reflex using transcutaneous electrical nerve stimulation, and the other experiment was performed to test the T‐reflex by Achilles tendon taps. The order of experiments was randomized for each subject. We asked the subjects to maintain the usual level of physical activity during the period between the two experiments.

**FIGURE 1:**
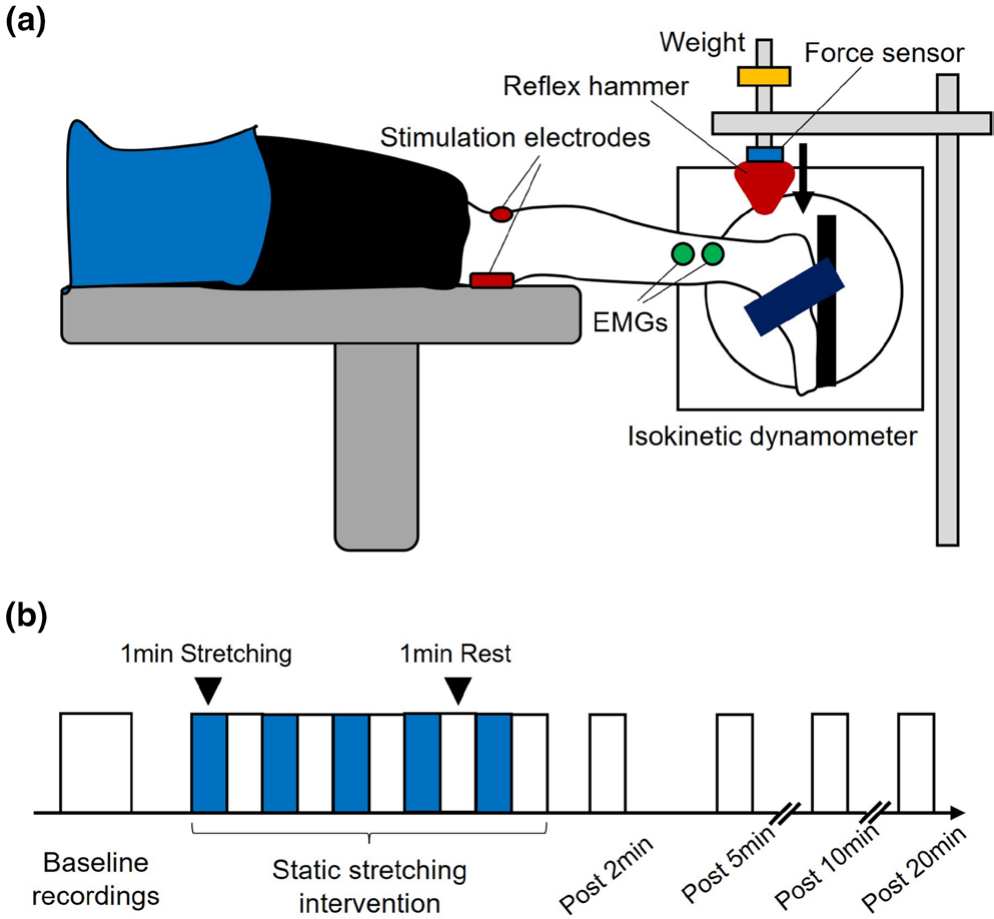
Experimental setup and procedure. (a) Experimental setup. Participants lay in a prone position with knees and hips fully extended. Electrode positions for transcutaneous electrical nerve stimulation and EMG recording from the soleus are shown. Tendon tap was performed using a reflex hammer dropped on the Achilles tendon from a constant height throughout the experiment. (b) Experimental procedure. Static stretching intervention comprised five 1‐min trials of static stretching with a 1‐min interval between each trial. Each block indicates five spinal reflexes (H‐reflex or T‐reflex) and one maximal M‐wave measurement. Measurements were performed at 0° of plantarflexion (white blocks) and at maximal dorsiflexion angle (blue blocks). H‐reflex and T‐reflex recordings were performed on different days.

Subjects were in the prone position with the hips and knees fully extended, and the right foot was attached to the footplate of an isokinetic dynamometer (Humac Norm CN77; CSMI, Stoughton, MA, USA) with a nonelastic strap (Figure 1). The center of the lateral malleolus of the right foot was visually aligned to the rotational axis of the dynamometer. In the present study, the ankle angle was defined as 0° when the footplate was perpendicular to the floor.

### 2.3 Static stretching

Static stretching involved five stretches lasting 1 min, with a 1‐min interval between each stretch. For each stretch trial, the right ankle was passively dorsiflexed from 20° of plantarflexion to maximal dorsiflexion and fixed there for 60 s, then moved to 0° of plantarflexion. The maximal angle of dorsiflexion was determined by passive ankle dorsiflexion testing before each stretching trial. The angle of maximal dorsiflexion was assessed by passively dorsiflexing the foot of the subject at an angular velocity of 1°/s from 20° of plantarflexion to the angle at which the subject first felt discomfort. The ankle joint angle was returned immediately to the plantarflexion position to avoid a stretching effect on ankle ROM. Each subject was asked to relax the lower limb and to not exert any voluntary resistance.

### 2.4 Surface electromyographic recordings

Surface electromyographic (EMG) signals were recorded from the SOL, medial gastrocnemius, lateral gastrocnemius, and tibialis anterior in the right lower leg. Ag‐AgCl electrodes (Vitrode F‐150S; Nihon Kohden, Tokyo, Japan) with an interelectrode distance of 20 mm were used for EMG acquisition from each muscle. Surface EMG electrodes for the SOL were attached distal and medial to the junction between the gastrocnemius and Achilles tendon. Surface EMG electrodes for the medial and lateral gastrocnemius were placed one‐third proximal to the line joining the calcaneus and the medial and lateral condyles of the tibia, respectively. For the tibialis anterior, electrodes were placed one‐third proximal to the line joining the lateral malleolus and the inferior edge of the patella. The amplifier was set to a gain of 1000‐fold with a bandpass filter between 5 Hz and 1 kHz (AB‐611J, Nihon Kohden). EMG signals and torque signals were simultaneously sampled at 4 kHz using an analog‐to‐digital converter (PowerLab; ADInstruments, Melbourne, Australia) and stored on a personal computer using software (LabChart 7, ADInstruments). Root‐mean‐square (RMS) values of EMG signals from the SOL, medial gastrocnemius, lateral gastrocnemius, and tibialis anterior during the passive ankle dorsiflexion test were determined for the initial 5° and final 5° of dorsiflexion, respectively (Mizuno, 2023). We ensured that each subject relaxed the lower legs during the passive ankle dorsiflexion test based on surface EMG recordings.

### 2.5 Transcutaneous electrical nerve stimulation

Transcutaneous electrical nerve stimulation was delivered to the posterior tibial nerve using a constant current electrical stimulator (DS7R; Digitimer Hertfordshire, UK). Rectangular stimulus pulse duration was set to 1 ms. The anode (5 cm × 5 cm) was placed on the patella. The cathode (diameter, 1 cm) was placed over the posterior tibial nerve at the popliteal fossa, which induced the largest H‐reflex response from the SOL.

### 2.6 Tendon taps

T‐reflexes in the SOL were evoked by tendon tap using a handmade reflex hammer (Takei Scientific Instruments, Niigata, Japan). The rubber‐tipped hammer was dropped on the Achilles tendon from a constant height (about 8–10 cm). At the beginning of measurement, the optimal dropping point and mass of the reflex hammer providing the largest T‐reflex response was identified. Consequently, the dropping point was 2–4 cm above the insertion of the Achilles tendon onto the calcaneus, and the mass of the hammer was adjusted by adding weight (100–300 g). The force sensor was loaded on the reflex hammer and recorded as trigger.

### 2.7 Experimental procedures

Subjects were instructed to relax completely during H‐reflex and T‐reflex recordings. Prior to the baseline recordings, the recruitment curves of muscle compound action potential (M‐wave) and H‐reflex from the SOL were collected. Stimulation intensity gradually increased until no further increase was observed in the M‐wave amplitude of the SOL, medial gastrocnemius, and lateral gastrocnemius using 1‐mA increments. Maximal M‐wave response (M_max_) and twitch torque of the plantarflexors were evoked by setting the supramaximal stimulus intensity to 20% above the maximum experimental measurements. Mean supramaximal intensities to evoke M_max_ for H‐reflex and T‐reflex experiments were 28.0 ± 8.9 mA and 28.6 ± 8.3 mA, respectively. Ten spinal reflexes (H‐reflex or T‐reflex) and 2 M_max_ values were collected at 0° of plantarflexion as baseline recordings. Five spinal reflexes (H‐reflex or T‐reflex) and 1 M_max_ value were evoked, and the sequence of stimulations was performed within 1 min and repeated during static stretches, during intervals between stretches, and at 2, 5, 10, and 20 min after completion of stretches. These measurements during intervals and at 2, 5, 10, and 20 min after 5‐min static stretching were performed at 0° of plantarflexion. H‐reflex and T‐reflex responses were evoked every 10 s. To ensure that test stimulus intensity was constant throughout the experiment, the size of the M‐wave response in the SOL evoked by test stimulation was maintained at 5% of M_max_ (Budini et al., 2018). The baseline size of the H‐reflex thus varied among subjects (33.3%–88.2% of M_max_). H‐reflex, T‐reflex, and M‐wave responses were measured as peak‐to‐peak amplitudes. Mean amplitudes of the H‐reflex and T‐reflex are expressed as a percentage of the M_max_ at each time point.

### 2.8 Statistical analysis

Data are provided as mean ± SD. The normality of data distribution was investigated using the Shapiro–Wilk test for all data. Since the normality of data was not observed in all cases, nonparametric statistical tests were used. Differences in dorsiflexion angles among five trials during static stretching were analyzed using the Friedman test. When a significant effect was found, the Wilcoxon signed‐rank test was performed as a post‐hoc test. Differences in RMS values between the initial and final 5° of dorsiflexion during the passive ankle dorsiflexion test were analyzed for each muscle using the Wilcoxon signed‐rank test. Changes in the amplitudes of the H‐reflex, T‐reflex, and M‐wave between the baseline values and each of 14 time points following the interventions were performed using the Friedman test. When a significant effect was found, the Wilcoxon signed‐rank test was performed as a post hoc test. Statistical significance was accepted for a value of *p* < 0.05, and *p* values for multiple comparisons were adjusted by Bonferroni correction. The effect size (*r*), which was calculated by the *z* value of the Wilcoxon signed‐rank test and sample size, was categorized as trivial (0–0.09), small (0.1–0.29), medium (0.3–0.5), or large (>0.5).

## 3 RESULTS

**FIGURE 2:**
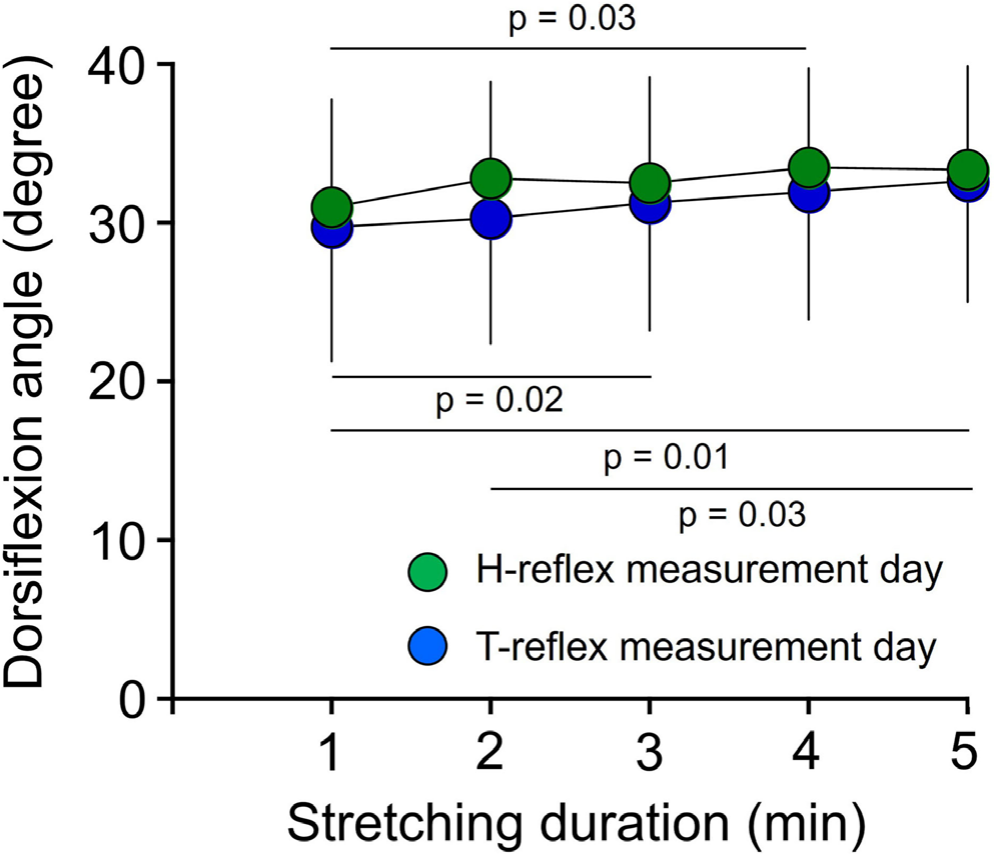
Stretching intensity and stretching duration. Static stretching intervention involved five repetitions of a 1‐min stretch at the maximal dorsiflexion angle. Values in each plot represent mean and SD.

A significant difference in dorsiflexion angle during static stretching was observed on the H‐reflex measurement day (*p* < 0.01) and T‐reflex measurement day (*p* < 0.01) according to the Friedman test (Figure 2). Effects of the duration maintaining a specific dorsiflexion angle during static stretching were observed in both measurements. For H‐reflex measurements, dorsiflexion angle was higher with the 4th stretch than with the 1st stretch (*p* = 0.03; *r* = 0.68). For T‐reflex measurements, dorsiflexion angle was higher with the 5th stretch than with the 1st (*p* = 0.01; r = 0.76) or 2nd stretch (*p* = 0.03; *r* = 0.68), and dorsiflexion angle was higher with the 3rd stretch than with the 1st (*p* = 0.02; *r* = 0.69).

RMS values for the initial 5° of SOL, medial gastrocnemius, lateral gastrocnemius, and tibialis anterior were 29.0 ± 14.6 μV, 29.5 ± 12.1 μV, 31.0 ± 11.3 μV, and 30.2 ± 11.6 μV, respectively. RMS values for the final 5° of SOL, medial gastrocnemius, lateral gastrocnemius, and tibialis anterior were 29.1 ± 14.1 μV, 30.0 ± 12.4 μV, 31.2 ± 11.9 μV, and 31.6 ± 13.2 μV, respectively. No significant changes in RMS values of any muscles were identified between the initial and final 5° of dorsiflexion during the passive ankle dorsiflexion test (*p* = 0.17–0.63; effect size = 0.11–0.32).

**FIGURE 3:**
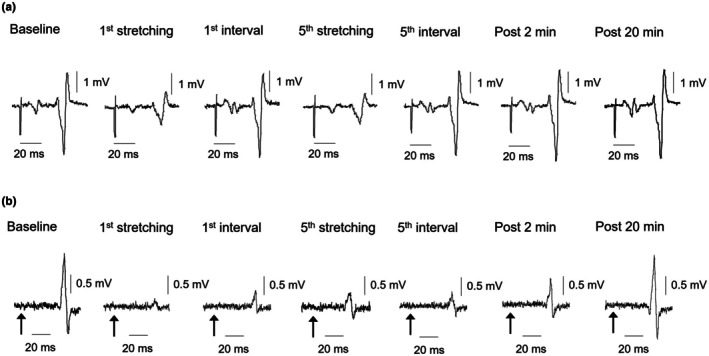
Typical waveforms of the H‐reflex (a) and T‐reflex (b) responses from a single participant. Black arrows indicate the timing of the Achilles tendon tap using the reflex hammer.

**FIGURE 4:**
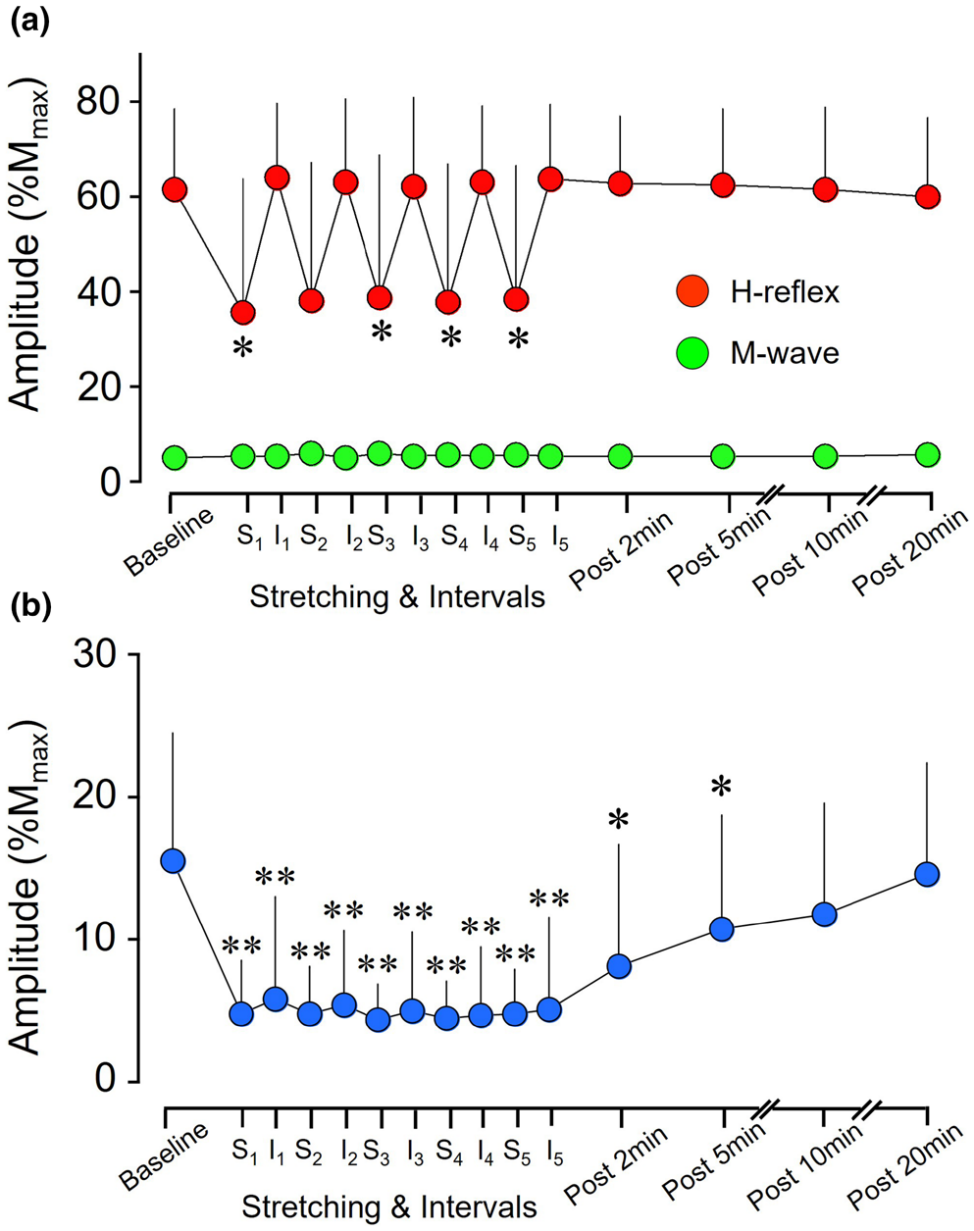
Modulations of the H‐reflex (a) and T‐reflex (b) amplitudes during and after static stretching intervention. Mean amplitudes of the H‐reflex and T‐reflex responses are normalized to M_max_ at each time point. S_1–5_: Number of 1‐min static stretches. I_1–5_: Number of the interval between static stretching interventions. Values in each plot represent mean and SD. *p < 0.05 versus baseline; **p < 0.01 versus baseline.

H‐reflex amplitudes were depressed 51.5%–55.2% from baseline during static stretching, but these depressions of the H‐reflex returned to baseline in each following interval (Figure 3a). A significant difference in H‐reflex amplitudes was observed according to the Friedman test (*p* < 0.01) (Figure 4a). H‐reflex amplitude was significantly lower at the 1st (*p* = 0.01; *r* = 0.74), 3rd (*p* = 0.04; *r* = 0.67), 4th (*p* = 0.04; *r* = 0.69) and 5th static stretches (*p* = 0.02; *r* = 0.72) compared to baseline. Moreover, no significant difference in M‐wave amplitude was detected according to the Friedman test (*p* = 0.14) (Figure 4a). The intensity of the test stimulus needed to evoke the H‐reflex response was thus maintained between measurements.

T‐reflex responses were depressed 71.2%–73.5% from baseline during static stretching, and these depressions of the T‐reflex were 58.8%–68.3% in each following interval (Figure 3b). A significant difference was observed in T‐reflex amplitudes using the Friedman test (*p* < 0.01) (Figure 4b). T‐reflex amplitude at each static stretch was significantly lower than that at baseline (*p* < 0.01 each; *r* = 0.87). T‐reflex amplitude at each interval was significantly lower than the amplitude at baseline (*p* < 0.01; *r* = 0.84–0.87). No significant difference in T‐reflex amplitude was observed between the 1st and 5th intervals (*p* = 0.36–0.73; *r* = 0.11–0.38). Regarding the recovery of T‐reflex responses after static stretching intervention, T‐reflex amplitudes at 2 min (*p* = 0.01; *r* = 0.79) and 5 min (*p* = 0.04; *r* = 0.69) after static stretching were significantly lower than that at baseline (Figure 4b). T‐reflex amplitude at 10 min (*p* = 0.15; *r* = 0.58) and 20 min (*p* = 0.68; *r* = 0.09) after static stretching did not differ significantly from that at baseline.

## 4 DISCUSSION

The purpose of the present study was to clarify the effect of static stretching durations on the modulation of muscle spindle sensitivity in the SOL muscle. The main finding was that no duration effect of static stretching was seen on modulations of H‐reflex or T‐reflex amplitudes in the SOL in the intervals following static stretching. These results did not support our hypothesis.

The present study demonstrated that stretching duration did not influence changes in T‐reflex excitability in the interval following each static stretch (Figure 4b). Both extra‐ and intrafusal muscle fibers are elongated during passive stretching, and the muscles then passively returned to their initial length, but some slack fibers remain in muscle spindles and cause a reduction in sensitivity (Gregory et al., 1987). Previous studies have indicated that recovery from T‐reflex depression requires 5–10 min following the cessation of static stretching (Budini et al., 2017, 2019). Although the present study was not able to identify to what extent muscle spindle sensitivity recovers within a 1‐min interval following each static stretch, long‐lasting depression of T‐reflex responses was maintained for at least 5 min after static stretching (Figure 4b). Moreover, modulation of the T‐reflex response is more likely to be attributable to reductions in muscle spindle sensitivity owing to the formation of slack in intrafusal fibers rather than to enhanced inhibition at the synaptic, cortical, or spinal levels. The present results therefore suggest that the duration of stretching does not influence the modulation of muscle spindle sensitivity by static stretching.

Previous studies have demonstrated considerable reductions in T‐reflex and stretch reflex excitability after static stretching (Avela et al., 1999; Budini et al., 2017, 2018, 2019; Guissard & Duchateau, 2006). For example, the T‐reflex amplitude of the SOL decreased 59.2% after 1 min of static stretching compared to the control condition (Budini et al., 2017), and 5 min of static stretching decreased the T‐reflex amplitude of the SOL by 57.6% compared to before stretching (Budini et al., 2019). Moreover, 10 min of static stretching decreased T‐reflex amplitude in the SOL by approximately 45% compared to the amplitude before stretching (Guissard & Duchateau, 2006). Given that a constant angle continues the stretch procedure, the results of these previous studies implied that the T‐reflex can only be inhibited up to a certain point, regardless of the duration of the stretch applied (Budini et al., 2019). Hence, the present study adopted an intermittent stretch protocol that involved 1 min of stretching performed five times with determination of the maximal angle of dorsiflexion in each stretching trial. Consequently, we showed that dorsiflexion angles increased with cumulative duration of stretching (Figure 2). The depression of T‐reflex responses from baseline was 58.8% at the 1st interval, but no further depression was observed in the 2nd to 5th intervals (61.9%–68.3% depression from baseline) (Figure 4b). This suggests that 1 min of static stretching at maximal dorsiflexion may achieve sufficient modulation of muscle spindle sensitivity in the SOL.

H‐reflex amplitude decreased from baseline during static stretching, and the H‐reflex recovered to baseline in the following interval (Figure 4a). Spinal reflex excitability is reportedly strongly inhibited during static stretching, and this inhibition disappears within several seconds after stretching (Budini et al., 2018; Funase et al., 2003; Obata et al., 2022). The H‐reflex response is modified by both pre‐ and postsynaptic factors influencing spinal motoneurons. The reduction of spinal reflex excitability during the lower intensity stretching would be attributed to presynaptic inhibition of the Ia afferents (Guissard et al., 2001; Guissard & Duchateau, 2006). Presynaptic inhibition is observed for only 300–400 ms after passive dorsiflexion, whereas postsynaptic depression persists for more than 10 s because postsynaptic depression is restricted to the homonymous muscle (Robertson & Koceja, 2003). A reduction in spinal reflex circuits is suggested to result from reductions in excitatory neural inputs from the Ia afferents onto the motoneurons, possibly due to decreased resting discharge of the muscle spindles via increased compliance of the muscle‐tendon unit (Avela et al., 1999). Moreover, corticospinal excitability in the SOL was facilitated up to 2 s after static stretching, then quickly returned to the baseline value (Budini et al., 2018). Other neural mechanisms may thus be involved in the modulation of spinal reflex excitability after static stretching as postsynaptic factors, such as recurrent inhibition, and afferent activity from cutaneous and joint receptors.

There were limitations in measuring the H‐reflex and T‐reflex in the present study. The sensitivity of the H‐reflex to facilitation and inhibition is related to the size of the test H‐reflex (Crone et al., 1987). To evoke the H‐reflex from the SOL in the present study, stimulation intensity was adjusted to obtain a value for which the H‐reflex responses were in the ascending limb and an M‐wave was visible (approximately 5% of M_max_). Regarding T‐reflex measurement, the reflex hammer was set to evoke the largest T‐reflex response from the SOL. Consequently, the sizes of the H‐reflex and T‐reflex responses at baseline were 61.5 ± 16.6% and 15.5 ± 8.9% of M_max_ (Figure 4). The difference in the size of H‐reflex and T‐reflex responses among subjects was thus considered one limitation when comparing the modulation of H‐reflex and T‐reflex amplitudes. Another limitation was the procedure for static stretching. For example, in comparisons of intermittent stretching (five 1‐min stretches with 15‐s intervals) and continuous stretching protocols (5‐min stretching), intermittent stretching had a greater effect than continuous stretching on muscle strength impairment and reduction of muscle activity in plantarflexors (Trajano et al., 2014). Hence, rest intervals between stretching may affect modulations in the spinal reflex circuits. A previous study recommended that more than three trials are needed to improve the accuracy of H‐reflex measurements (Doguet & Jubeau, 2014). The present study adopted five 1‐min stretches with 1‐min intervals (i.e., equal stretching and interval durations) (Figure 1). This was because the T‐reflex and H‐reflex measurements needed 1 min to evoke the five responses to improve reliability.

In conclusion, no effect of the duration of static stretching on modulation of muscle spindle sensitivity in the SOL was observed. The present study investigated the modulation of H‐reflex and T‐reflex amplitudes with five static stretches lasting 1 min each. The depression of the H‐reflex amplitude during static stretching recovered in the interval following each stretch. T‐reflex amplitude was strongly depressed after 1‐min static stretching, but no further depression was observed regardless of the duration or intensity of static stretching. These findings suggest that 1‐min static stretching with maximal dorsiflexion angle results in sufficient modulation of muscle spindle sensitivity of the SOL.

## AUTHOR CONTRIBUTIONS

A.S. conceived and designed research, performed the experiments, analyzed data, interpreted results of experiments, prepared figures, drafted manuscript, edited and revised manuscript, and approved final version of the manuscript. T.M. conceived and designed research, interpreted results of experiments, edited and revised manuscript, and approved final version of the manuscript.

## FUNDING INFORMATION

The present study was supported by Tobemaki Scholarship Foundation (24‐JC‐001) to AS.

## CONFLICT OF INTEREST STATEMENT

The authors declare no conflicts of interest.

## ETHICS STATEMENT

The ethics review committee for experimental research involving human subjects at Kyushu Sangyo University approved the experimental protocols (approval no. 2022‐0010), which were conducted in accordance with the guidelines of the Declaration of Helsinki.

## Data Availability

Data will be made available upon reasonable request to the corresponding author.

## References

[phy270538-bib-0001] Avela, J. , Kyröläinen, H. , & Komi, P. V. (1999). Altered reflex sensitivity after repeated and prolonged passive muscle stretching. Journal of Applied Physiology (1985), 86, 1283–1291. 10.1152/jappl.1999.86.4.1283 10194214

[phy270538-bib-0002] Behm, D. G. , Blazevich, A. J. , Kay, A. D. , & McHugh, M. (2016). Acute effects of muscle stretching on physical performance, range of motion, and injury incidence in healthy active individuals: A systematic review. Applied Physiology, Nutrition, and Metabolism, 41, 1–11. 10.1139/apnm-2015-0235 26642915

[phy270538-bib-0003] Behm, D. G. , & Chaouachi, A. (2011). A review of the acute effects of static and dynamic stretching on performance. European Journal of Applied Physiology, 111, 2633–2651. 10.1007/s00421-011-1879-2 21373870

[phy270538-bib-0004] Budini, F. , Christova, M. , Gallasch, E. , Kressnik, P. , Rafolt, D. , & Tilp, M. (2018). Transient increase in cortical excitability following static stretching of plantar flexor muscles. Frontiers in Physiology, 9, 530. 10.3389/fphys.2018.00530 29942261 PMC6004398

[phy270538-bib-0005] Budini, F. , Gallasch, E. , Christova, M. , Rafolt, D. , Rauscher, A. B. , & Tilp, M. (2017). One minute static stretch of plantar flexors transiently increases H reflex excitability and exerts no effect on corticospinal pathways. Experimental Physiology, 102, 901–910. 10.1113/EP086374 28585766

[phy270538-bib-0006] Budini, F. , Kemper, D. , Christova, M. , Gallasch, E. , Rafolt, D. , & Tilp, M. (2019). Five minutes static stretching influences neural responses at spinal level in the background of unchanged corticospinal excitability. Journal of Musculoskeletal & Neuronal Interactions, 19, 30–37. 30839301 PMC6454261

[phy270538-bib-0007] Crone, C. , Hultborn, H. , Jespersen, B. , & Nielsen, J. (1987). Reciprocal Ia inhibition between ankle flexors and extensors in man. The Journal of Physiology, 389, 163–185. 3681725 10.1113/jphysiol.1987.sp016652 PMC1192076

[phy270538-bib-0008] Doguet, V. , & Jubeau, M. (2014). Reliability of H‐reflex in vastus lateralis and vastus medialis muscles during passive and active isometric conditions. European Journal of Applied Physiology, 114, 2509–2519. 10.1007/s00421-014-2969-8 25113094

[phy270538-bib-0009] Funase, K. , Higashi, T. , Sakakibara, A. , Tanaka, K. , Takemochi, K. , Ogahara, K. , & Iwanaga, R. (2003). Neural mechanism underlying the H‐reflex inhibition during statix muscle stretching. Advances in Exercise and Sports Physiology, 9, 119–127.

[phy270538-bib-0010] Gregory, J. E. , Morgan, D. L. , & Proske, U. (1987). Changes in size of the stretch reflex of cat and man attributed to aftereffects in muscle spindles. Journal of Neurophysiology, 58, 628–640. 10.1152/jn.1987.58.3.628 2958607

[phy270538-bib-0011] Guissard, N. , & Duchateau, J. (2006). Neural aspects of muscle stretching. Exercise and Sport Sciences Reviews, 34, 154–158. 10.1249/01.jes.0000240023.30373.eb 17031252

[phy270538-bib-0012] Guissard, N. , Duchateau, J. , & Hainaut, K. (1988). Muscle stretching and motoneuron excitability. European Journal of Applied Physiology and Occupational Physiology, 58, 47–52. 10.1007/bf00636602 3203674

[phy270538-bib-0013] Guissard, N. , Duchateau, J. , & Hainaut, K. (2001). Mechanisms of decreased motoneurone excitation during passive muscle stretching. Experimental Brain Research, 137, 163–169. 11315544 10.1007/s002210000648

[phy270538-bib-0014] Kay, A. D. , & Blazevich, A. J. (2012). Effect of acute static stretch on maximal muscle performance: A systematic review. Medicine and Science in Sports and Exercise, 44, 154–164. 10.1249/MSS.0b013e318225cb27 21659901

[phy270538-bib-0015] McNeil, C. J. , Butler, J. E. , Taylor, J. L. , & Gandevia, S. C. (2013). Testing the excitability of human motoneurons. Frontiers in Human Neuroscience, 7, 152. 10.3389/fnhum.2013.00152 23630483 PMC3633937

[phy270538-bib-0016] Mizuno, T. (2023). Combined static stretching and electrical muscle stimulation induce greater changes in range of motion, passive torque, and tendon displacement compared with static stretching. Sports, 11, 10. 10.3390/sports11010010 36668714 PMC9864422

[phy270538-bib-0017] Obata, H. , Kim, G. , Ogawa, T. , Sekiguchi, H. , & Nakazawa, K. (2022). Effect of long‐term classical ballet dance training on postactivation depression of the soleus Hoffmann‐reflex. Motor Control, 26, 169–180. 10.1123/mc.2021-0079 34986460

[phy270538-bib-0018] Proske, U. , Morgan, D. L. , & Gregory, J. E. (1993). Thixotropy in skeletal muscle and in muscle spindles: A review. Progress in Neurobiology, 41, 705–721. 10.1016/0301-0082(93)90032-n 8140258

[phy270538-bib-0019] Robertson, C. T. , & Koceja, D. M. (2003). Post‐activation depression of the soleus H‐reflex in the elderly. Electromyography and Clinical Neurophysiology, 43, 103–111. 12661135

[phy270538-bib-0020] Trajano, G. S. , Nosaka, K. , L, B. S. , & Blazevich, A. J. (2014). Intermittent stretch reduces force and central drive more than continuous stretch. Medicine and Science in Sports and Exercise, 46, 902–910. 10.1249/MSS.0000000000000185 24121249

[phy270538-bib-0021] Witvrouw, E. , Danneels, L. , Asselman, P. , D'Have, T. , & Cambier, D. (2003). Muscle flexibility as a risk factor for developing muscle injuries in male professional soccer players. A prospective study. American Journal of Sports Medicine, 31, 41–46. 10.1177/03635465030310011801 12531755

[phy270538-bib-0022] Woods, K. , Bishop, P. , & Jones, E. (2007). Warm‐up and stretching in the prevention of muscular injury. Sports Medicine, 37, 1089–1099. 10.2165/00007256-200737120-00006 18027995

